# Recurrent Ischemic and Hemorrhagic Stroke in Cameroon: A Case-Control Study

**DOI:** 10.1155/2021/9948990

**Published:** 2021-06-15

**Authors:** Jaurès Kamgang, Francklin Tétinou, Yvan Zolo, Chee Yang Tan, Christian Wambo, Emerancienne J. N. Fongang, Ulrick Sidney Kanmounye

**Affiliations:** ^1^Faculty of Medicine, Higher Institute of Health Sciences, Université des Montagnes, Bangangté, Cameroon; ^2^Research Department, Association of Future African Neurosurgeons, Yaounde, Cameroon; ^3^Faculty of Health Sciences, University of Buea, Buea, Cameroon; ^4^Faculty of Medicine, University of Malaya, Kuala Lumpur, Malaysia; ^5^Faculty of Medicine and Pharmaceutical Sciences, University of Douala, Douala, Cameroon; ^6^Neurosurgery Unit, Laquintinie Hospital, Douala, Cameroon

## Abstract

**Introduction:**

Stroke recurrence accounts for a great percentage of catastrophic complications, yet no comprehensive study has analyzed the factors associated with stroke recurrence in Cameroon. We carried out this case-control study to better understand the factors associated with the stroke recurrence in Cameroon.

**Methods:**

We collected sociodemographic, clinical, neuroimaging, laboratory, and therapeutic data of eligible patients who consulted the neurology and cardiology department of the Yaounde Central Hospital in Cameroon. We included all patients at least five years removed from their first stroke event who consulted the authors' institution as of January 15, 2019. Wilcoxon signed-rank and Fisher's exact tests were used. Also, a Cox regression model was used to identify confounders.

**Results:**

We recruited 100 patients; seven out of ten patients had hypertension, while six out of 10 had a sedentary lifestyle. Half of the patients consumed alcohol regularly, while one patient out of five had diabetes. Most patients presented with their first stroke event, and a quarter had a stroke recurrence. Stroke recurrence was associated with right handedness (OR = 0.23, 95% CI = 0.16–0.33), congestive heart failure (OR = 3.45, 95% CI = 1.16–10.28), gout (OR = 4.34, 95% CI = 1.09–18.09), dysarthria (OR = 4.34, 95% CI = 1.30–14.54), and facial palsy (OR = 3.96, 95% CII = 1.49 – 10.51), as well as modifiable factors such as elevated abdominal circumference (*P* < 0.01), systolic blood pressure (*P* < 0.01), blood glucose level (*P*I <I 0.01), LDL cholesterol (*P* < 0.01), and triglyceride levels (*P* < 0.01). The mulitvariable regression model only identified laterality (*B* = –1.48, *P* = 0.04) as a statistically significant explanatory varibale for stroke recurrence.

**Conclusion:**

We mapped the landscape of recurrent strokes in Cameroon. There is a need to evaluate the causes of suboptimal drug adherence rates and both the role and adherence to nonpharmacologic interventions.

## 1. Introduction

Strokes are the second leading cause of death globally; nearly 16 million new cases and 5.7 million deaths are registered each year, 87% of which are in low- and middle-income countries (LMICs) [1]. In Cameroon, the prevalence rate of stroke is 7.3%, and its mortality rates are 26.7% during the first month and 31.7% in the first three months after the stroke event [2, 3].

Stroke recurrence is one of the most frequent and feared complications of a stroke [4]. Its prevalence varies widely. For example, in England, it is estimated at 5.6% over the first 12 months following an index stroke event [5], while in Ireland, it is almost twice the English rates (10.8%) [6]. In Cameroon, the stroke recurrence rate has been estimated at 14.5% [7]. There are several factors incriminated in stroke recurrence. These include a history of transient ischemic attack, daily alcohol consumption, smoking, age, atrial fibrillation, and diabetes [5, 8, 9]. Despite progress in preventing and treating these factors, stroke recurrence is very prevalent and deadly [7]. Recurrent strokes have higher mortality rates than index stroke events causing deaths in more than 30.6% of Senegalese patients in the first year alone [10] and 43% of Cameroonians [7].

No study has analyzed the link between sociodemographic and clinical factors on stroke recurrence in Cameroon. We carried out this case-control study to better understand the factors associated with the recurrence of stroke in Cameroon.

## 2. Materials and Methods

This case-control study was conducted in the neurology and cardiology departments of the Yaounde Central Hospital, Cameroon, a tertiary facility in Cameroon's capital.

### 2.1. Ethics

The institutional review boards of Université des Montagnes, Bangangté, Cameroon (Ref: 2019/212/UdM/PR/CIE), and the Yaounde Central Hospital (Ref: 0098/19/AR/MINSANTE/SG/DHCY/CM/SM) approved the study as part of the first author's (JK) medical thesis.

### 2.2. Definition of Terms

For this case-control study, recurrent stroke was defined as “clinical evidence of the sudden onset of a new focal neurological deficit with no apparent cause other than that of vascular origin (i.e., the deficit could not be ascribed to a concurrent acute illness, epileptic seizure, or toxic effect) occurring at any time after a (documented) index stroke” [4]. Patients who had suffered a recurrent stroke were defined as cases, while patients who had suffered their index stroke were defined as controls.

Medication adherence rates were defined as “the sum of the days' supply of drug therapy over (unit time) observed” [11]. Drug adherence was then classified into complete adherence (95–100% adherence rate), intermittent underexposure (>0% but <95% adherence rate), and permanent discontinuation (0% adherence rate) [12].

### 2.3. Data Collection

All patients at least five years (60 months) removed from their first stroke event who consulted at Yaounde Central Hospital as of January 15, 2019, were eligible. Patients without radiological confirmation of stroke were excluded from the study. The sampling was consecutive, and a minimum sample size of 48 was estimated using Cochrane's formula. The hospital prevalence of recurrent stroke in Cameroon was extracted from the work of Lekoubou et al. [7], i.e., 14.5%. Sociodemographic (age, gender, marital status, level of education, and profession), clinical (reason for consultation, vascular risk factors, comorbidities, time between the 1st episode of stroke and recurrence, and physical examination), neuroimaging, laboratory, and therapeutic data were extracted from patient charts. The authors contacted eligible patients by phone and during neurology clinics. Consent was sought during the index contact, and patients who declined consent were excluded from the study.

### 2.4. Data Analysis

The authors designed a five-part data collection tool administered by the first author (JK). The five parts collected sociodemographic (sex, age, marital status, level of education, urban/rural dwelling, and profession), clinical (history and presentation), workup (head computed tomography scan), therapeutic (medical and physical therapy), and follow-up data. Descriptive summary analysis was conducted for quantitative data (mean and standard deviation or median and interquartile range) and qualitative data (frequency and percentage). Wilcoxon signed-rank and Fisher's exact tests evaluated bivariable relationships between stroke recurrence status and independent variables (sociodemographic, clinical, and radiological data).

Moreover, adjusted odds ratios and *P*values were calculated for variables showing statistical significance in the bivariable analysis. Additionally, the median time from the index stroke to the second stroke was calculated for qualitative variables showing statistical significance during the bivariable analysis. These variables were then integrated into a 1000-sample bootstrap Cox regression model.

## 3. Results

We recruited 100 stroke patients aged 29.6 ± 11.0 years. Most patients were male (57.0%), married (64.0%), urban dwellers (64.0%), and right-handed (96.0%). 71 patients (71.0%) had hypertension, 59.0% had a sedentary lifestyle, 51.0% consumed alcohol regularly, 21.0% had diabetes, 17.0% had heart failure, and 17.0% consumed tobacco. Only 27.0% had a family history of stroke (Table 1).

Most patients (75.0%) had had a single stroke event, and 25.0% had a recurrent stroke, with one of them presenting with his third stroke event. Twenty-six patients (26.0%) had facial palsy, 19.0% had aphasia, 17.0% had alternating hemiplegia, 13.0% had dysarthria, 10% presented signs of intracranial hypertension, 3.0% had cerebellar syndrome, and 3.0% had signs of meningeal irritation.

Ischemic strokes were in the superficial (32.0%, *n* = 32), deep (15.0%, *n* = 15), and large (10.0%, *n* = 10) middle cerebral artery (MCA) territories, while nine patients (9.0%) had posterior cerebral artery (PCA) strokes. Hemorrhagic strokes were lenticular (5.0%), and four patients (4.0%) had a concomitant intraventricular hemorrhage. Five patients (5.0%) had another stroke in the same location as the index stroke event.

**Figure 1:**
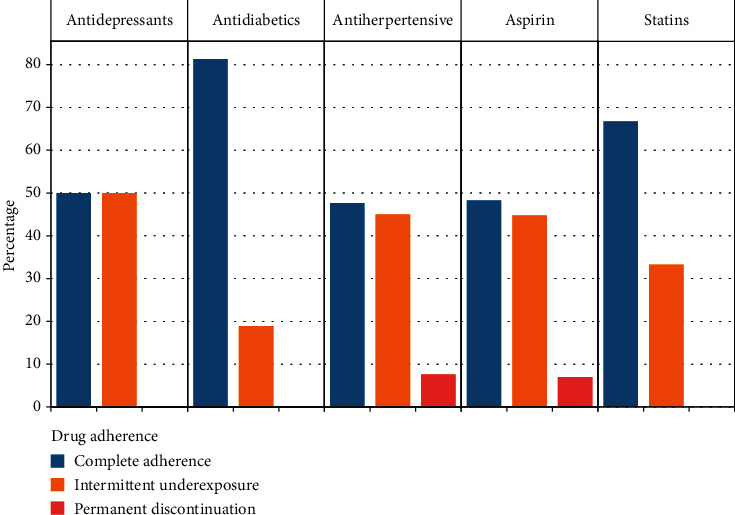
Medication adherence of Cameroonian stroke patients.

Most patients were taking antihypertensive medications (80.0%), 27.0% were taking acetylsalicylic acid, 17.0% were taking antidiabetics, 12.0% were taking statins, and 4.0% were taking antidepressants. In general, medication adherence rates were suboptimal (Figure 1). In addition, 18.0% of patients were treated with kinesiotherapy.

Stroke recurrence was associated with right-handedness (OR = 0.23; 95% CI = 0.16–0.33; *P* = 0.02), congestive heart failure (OR = 3.45; 95% CI = 1.16–10.28; *P* = 0.03), gout (OR = 4.44; 95% CI = 1.09–18.09; *P* = 0.04), dysarthria (OR = 4.34; 95% CI = 1.30–14.54; *P* = 0.02), and facial palsy (OR = 3.96; 95% CI = 1.49–10.51; *P* = 0.01). The association between medication observance and stroke recurrence was not statistically significant (Table 2).

The median time from the index stroke event to the second stroke was 16.0 (95% CI = 3.36–28.64) months in right-handed patients, 19.0 (95% CI = 3.76–34.25) months in patients with heart failure, and 15.0 (95% CI = 0.78–29.22) months in patients with hypertension. After running the bootstrap Cox regression model, laterality was statistically significant (*B* = -1.48, *P* = 0.04). However, hypertension (*B* = 0.57, *P* = 0.30) and congestive heart failure (*B* = 0.60, *P* = 0.20) were not.

There were statistically significant differences in the abdominal circumference, heart rate, respiratory rate, systolic blood pressure, blood glucose level, hemoglobin concentration, platelet count, LDL cholesterol, triglycerides, and intraventricular hemorrhage of patients with recurrent and those without recurrent strokes (*P* = <0.01) (Table 3).

## 4. Discussion

In this study, we investigated the factors associated with stroke recurrence in Cameroon. Most patients presented with their first stroke event, and a quarter of them had a stroke recurrence. Stroke recurrence was associated with modifiable factors such as elevated abdominal circumference, SBP, blood glucose level, LDL cholesterol, and triglyceride levels. Additionally, stroke recurrence was associated with laterality, congestive heart failure, gout, dysarthria, and facial palsy.

Recrudescence of old stroke deficits is common but underdiagnosed and can be confused with stroke recurrence. In our study, only 5.0% of patients had a stroke event in the same territory as the index stroke (confirmed radiologically). These findings suggest that recrudescence was very low in this population. We equally found that increased SBP and DBP were associated with stroke recurrence during bivariable analysis. However, SBP was the only statistically significant blood pressure measurement once the odds ratios were adjusted. This result indicates that DBP was a confounder. Our results are consistent with previous studies that have demonstrated that increased SBP increases the risk of stroke recurrence [13–17]. SBP measurement during the first 24 hours of stroke is a prognostic factor of stroke recurrence and has been used to identify high-risk patients [18]. The Perindopril Protection against Recurrent Stroke Study (PROGRESS) found that lowering SBP after a stroke reduced the risks of recurrent stroke among both hypertensive and nonhypertensive patients [19]. Another study showed that intensive lowering of blood pressure reduced stroke recurrence and recommended that the blood pressure target of less than 130/80 mmHg for secondary stroke prevention [20].

Diabetes was not associated with stroke recurrence in our study; however, increased casual blood glucose was statistically associated with stroke recurrence. This suggests that blood glucose control is just as important as diabetes status in preventing recurrent stroke. This finding highlights the necessity for blood glucose management and taking into consideration the low medication adherence rates. The results should be used to advocate for individual-level and public health measures that promote adherence. Our findings contradict several studies that have found a history of diabetes as an independent risk factor for an isolated stroke event and stroke recurrence. An HbA1c level ≥6.1% at admission is an independent predictor for stroke recurrence, and the risk of recurrent stroke increases with increasing HbA1c values [21–25]. In this study, we used the casual blood sugar level; however, fasting blood sugar is a better predictor of neurological outcome than casual blood sugar and HbA1c following an acute stroke event [26]. It is unclear if fasting blood glucose is a better predictor of stroke recurrence than casual blood glucose and HbA1c in this population.

Elevated LDL cholesterol and triglyceride levels were independently associated with stroke recurrence in this study. Elevated LDL is an independent predictor of stroke recurrence, and statins reduce LDL cholesterol levels and their associated recurrence risk [27]. Most patients prescribed statins in this study were observant. However, we did not find evidence that statin observance was statistically associated with decreased stroke recurrence risk. This finding warrants further investigation. Possible explanations include the dosage and LDL cholesterol target. Other lipid parameters such as triglycerides can be targeted to reduce the risk of stroke recurrence [28] and they should be studied in our setting.

Recurrent strokes often affect the same brain hemisphere and location as the index stroke [5]. This tropism for the injured brain is due to altered anatomy and physiology of the injured brain [15]. An index stroke damages the cerebral blood vessels making them prone to subsequent strokes [29]. Also, risk factors that alter the function and lumen of blood vessels such as atherosclerosis increase the probability of recurrence in the same territory as the index stroke [30]. Due to the complexity of stroke recurrence, multiple studies are underway to understand better the factors surrounding lateralization of stroke recurrence [5].

We recognize a few limitations to this study. First, the study was retrospective and, therefore, prone to recall bias. We tried to mitigate this bias by carefully designing the research questions. Next, we did not evaluate adherence to nonpharmacologic measures. Given the high prevalence of stroke recurrence and its association with factors amenable to nonpharmacologic interventions, evaluating adherence to these nonpharmacologic interventions would have given a comprehensive understanding of stroke recurrence in Cameroonian patients. In addition, laterality was heavily skewed in favor of right-handedness and right-handedness seemed to be protective of stroke recurrence. Hence, our results on the role of laterality on stroke recurrence should be interpreted with caution. 

## 5. Conclusions

We set out to understand why stroke recurrence was very high in our cohort, hypothesizing that some of the variables had a greater impact on recurrence than the others. Our study revealed insights into the landscape of recurrent strokes in Cameroon. First, they are prevalent and are associated with multiple modifiable risk factors. However, Cameroonian patients have suboptimal drug adherence rates. Future studies should evaluate the causes of suboptimal drug adherence rates and both the role and adherence to nonpharmacologic interventions. We will use the information from this study to influence department and ministry of health actions in the form of programs to improve medication adherence and research to evaluate how increased medication adherence will decrease the stroke risk.

## Figures and Tables

**Table 1:** Sociodemographic characteristics of Cameroonian stroke patients.

Characteristic	Frequency (percentage)
Sex	
Female	57 (57.0)
Male	43 (43.0)

Marital status	
Married	64 (64.0)
Widow	31 (31.0)
Single	2 (2.0)
Divorced	2 (2.0)

Level of education	
Secondary education	40 (40.0)
Primary education	25 (25.0)
Tertiary education	19 (19.0)
No formal education	15 (15.0)

Residency location	
Urban	64 (64.0)
Rural	35 (35.0)

Profession	
Civil servant	31 (31.0)
Unemployed	29 (29.0)
Private formal sector	19 (19.0)
Private informal sector	16 (16.0)

Laterality	
Right handed	96 (96.0)
Left handed	4 (4.0)

Personal history	
Sedentary lifestyle	51 (51.0)
Alcohol consumption	49 (49.0)
Hypertension	29 (29.0)
Diabetes	21 (21.0)
Congestive heart failure	17 (17.0)
Tobacco consumption	17 (17.0)
Gout	9 (9.0)
Chronic kidney disease	5 (5.0)
Malignancy	2 (2.0)
Family history of stroke	27 (27.0)

**Table 2:** Fisher's exact and odds ratios of sociodemographic, clinical, and therapeutic factors associated with stroke recurrence.

Factor	Odds ratio	95% confidence interval	*P*value
Male	0.68	0.27–1.73	0.49
Urban dwelling	1.04	0.40–2.67	0.94
Right handedness	0.23	0.16–0.33	0.02^*∗*^

Personal history			
Alcohol consumption	2.26	0.89–5.77	0.11
Tobacco consumption	1.81	0.59–5.54	0.36
Sedentary lifestyle	1.28	0.50–3.28	0.65
Chronic kidney disease	0.73	0.08–6.85	0.98
Congestive heart failure	3.45	1.16–10.28	0.03^*∗*^
Diabetes	2.21	0.79–6.20	0.16
Gout	4.44	1.09–18.09	0.04^*∗*^
Hypertension	1.40	0.50–3.97	0.62
Family history of stroke	1.34	0.50–3.60	0.61

Presenting signs			
Alternating hemiplegia	0.91	0.27–3.09	0.98
Aphasia	0.30	0.06–1.39	0.14
Cerebellar syndrome	1.61	0.14–18.66	0.57
Dysarthria	4.34	1.30–14.54	0.02^*∗*^
Facial palsy	3.96	1.49–10.51	0.01^*∗*^
Intracranial hypertension	0.31	0.04–2.54	0.44
Meningeal syndrome	0.74	0.66–0.84	0.57

Medication observance			
Antidepressant	N/A	N/A	N/A
Antidiabetic	3.20	0.23–45.19	0.55
Antihypertensive	0.64	0.24–1.72	0.47
Aspirin	0.79	0.10–6.50	0.98
Statin	0.88	0.67–1.14	0.98

**Table 3:** Wilcoxon ranked sum comparison of quantitative variables between Cameroonian patients with recurrent stroke and those with a first-time stroke event.

Quantitative variable	Test statistic	*P*value	Adjusted *P*value
Clinical parameters			
Age	−0.19	0.80	0.98
Abdominal circumference	9.14	<0.01^*∗∗*^	<0.01^*∗∗*^
Glasgow Coma Scale	1.22	0.09	0.98
Heart rate	3.87	<0.01^*∗∗*^	<0.01^*∗∗*^
Respiratory rate	12.30	<0.01^*∗∗*^	<0.01^*∗∗*^
Diastolic blood pressure	−2.05	<0.01^*∗∗*^	0.56
Systolic blood pressure	4.75	<0.01^*∗∗*^	<0.01^*∗∗*^

Laboratory workup			
Blood glucose	5.17	<0.01^*∗∗*^	<0.01^*∗∗*^
Hemoglobin	12.20	<0.01^*∗∗*^	<0.01^*∗∗*^
Platelet count	8.48	<0.01^*∗∗*^	<0.01^*∗∗*^
HDL cholesterol	1.86	0.01^*∗*^	0.98
LDL cholesterol	8.89	<0.01^*∗∗*^	<0.01^*∗∗*^
Triglycerides	6.91	<0.01^*∗∗*^	<0.01^*∗∗*^

Radiological			
Number of ventricles inundated	8.11	<0.01^*∗∗*^	<0.01^*∗∗*^

## Data Availability

The datasets generated and analyzed during the current study are not publicly available due to a restriction by Université des Montagnes but are available from the corresponding author on reasonable request.
